# All-Atom Molecular Dynamics Investigations on the Interactions between D2 Subunit Dopamine Receptors and Three ^11^C-Labeled Radiopharmaceutical Ligands

**DOI:** 10.3390/ijms23042005

**Published:** 2022-02-11

**Authors:** Sanda Nastasia Moldovean, Diana-Gabriela Timaru, Vasile Chiş

**Affiliations:** 1 Faculty of Physics, Babeş-Bolyai University, 400084 Cluj-Napoca, Romania; nastasia.moldovean@ubbcluj.ro (S.N.M.); diana.timaru@stud.ubbcluj.ro (D.-G.T.); 2 Biomolecular Modeling and Computational Spectroscopy Laboratory, Institute for Research, Development and Innovation in Applied Natural Sciences, Babeş-Bolyai University, 400327 Cluj-Napoca, Romania

**Keywords:** dopamine receptors, D2 subunit, molecular docking, molecular dynamics, interaction energies, ligand binding

## Abstract

The D2 subunit dopamine receptor represents a key factor in modulating dopamine release. Moreover, the investigated radiopharmaceutical ligands used in positron emission tomography imaging techniques are known to bind D2 receptors, allowing for dopaminergic pathways quantification in the living human brain. Thus, the biophysical characterization of these radioligands is expected to provide additional insights into the interaction mechanisms between the vehicle molecules and their targets. Using molecular dynamics simulations and QM calculations, the present study aimed to investigate the potential positions in which the D2 dopamine receptor would most likely interact with the three distinctive synthetic 11C-labeled compounds (raclopride (3,5-dichloro-N-[[(2S)-1-ethylpyrrolidin-2-yl]methyl]-2-hydroxy-6-methoxybenzamide)—RACL, FLB457 (5-bromo-N-[[(2S)-1-ethylpyrrolidin-2-yl]methyl]-2,3-dimethoxybenzamide)—FLB457 and SCH23390 (R(+)-7-Chloro-8-hydroxy-3-methyl-1-phenyl-2,3,4,5-tetrahydro-1H-3-benzazepine)—SCH)), as well as to estimate the binding affinities of the ligand-receptor complexes. A docking study was performed prior to multiple 50 ns molecular dynamics productions for the ligands situated at the top and bottom interacting pockets of the receptor. The most prominent motions for the RACL ligand were described by the high fluctuations of the peripheral aliphatic -CH3 groups and by its C-Cl aromatic ring groups. In good agreement with the experimental data, the D2 dopamine receptor-RACL complex showed the highest interacting patterns for ligands docked at the receptor’s top position.

## 1. Introduction

Dopamine (DA) is a catecholamine neurotransmitter [1] and its receptors are known to play a key role in neuronal signal transfer and processes such as reward, addiction, control of coordinated movements, glutathione metabolism (and the energetic metabolism of neurons), and hormonal secretion [2,3,4]. However, if dysregulations in the dopaminergic system occur, these may lead to severe disorders such as schizophrenia, Parkinson’s disease, attention deficit hyperactivity disorder, Tourette’s syndrome, depression, or nausea and vomiting [1].

Gene-cloning procedures of DA receptors allow for a better characterization of DA receptor subtypes (D1–D5), as well as their disposability throughout the central nervous system (CNS) and their role in specific disorders. Furthermore, experimental approaches led to a better understanding of the D1 and D2 receptors from a functional point of view. These studies allowed for a better characterization concerning D1DR and D2DR subunit behavior, as well as their interactions with other neurotransmitters [3,4].

The D2 dopamine receptor (encoded D2DR) is the main target for antipsychotic drugs or for drugs used in Parkinson’s disease treatment. D2DR acts as an autoreceptor and represents an essential factor in modulating DA release [2]. However, most drugs targeting D2DR can cause serious brain-level damage and have life-threatening side effects on movement control, emotional behavior, and mental health.

**Figure 1:**
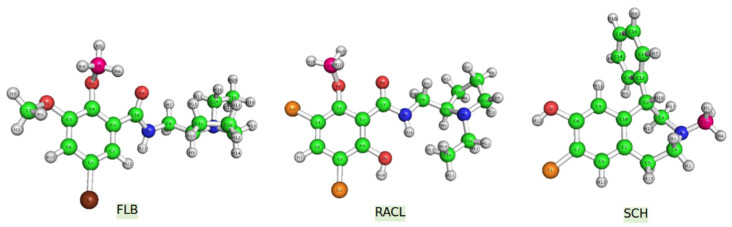
PCM (water)-B3LYP/6–311 + G (d,p) optimized molecular structures of the investigated ligands (the ^11^C atoms are illustrated in pink).

D2DR antagonists were initially developed to prevent hallucinations and delusions affecting people suffering from schizophrenia. Additionally, new D2DR antagonists can be used as tracers in positron emission tomography (PET). A proper example would be raclopride (Figure 1), a synthetic compound used for in vitro (autoradiography) techniques as well as in vivo (PET scans) imaging when labeled with radioisotopes (e.g., with ^11^C) [5]. The diagnosis of movement disorders and keeping Huntington’s disease under surveillance are main examples of raclopride usage. RACL is also helpful in measuring the potency and neurotoxicity of dopaminergic drugs [6].

Another well-known antagonist with a high affinity for D2DR in vitro is FLB457 (Figure 1). An experimental study [7] revealed that this molecule has a high binding affinity to D2 and D3 dopamine subunits in vitro, and it was also demonstrated that this compound does not only bind at the striatum level but in several extrastriatal brain regions too [7,8,9].

Halobenzazepine, also known as SCH23290 (Figure 1), is a synthetic compound with high affinity for the D1DR subunit. Despite this greater specificity for D1DR, several studies [10] have demonstrated that SCH also antagonizes processes (such as locomotion in control rats) induced by other compounds with a direct effect on the D2 dopamine receptor sites. Further research must be considered to determine whether dopaminergic compounds are essential for the SCH ligand’s effect on D2DR [10].

**Figure 2:**
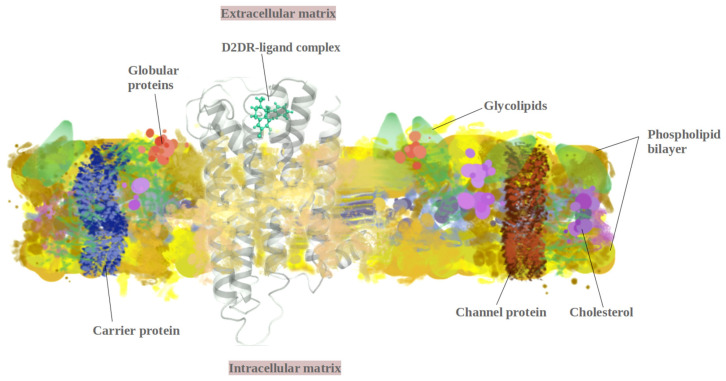
D2DR membrane protein structure.

Studies using molecular dynamics (MD) [11] simulations covered many aspects of D2DR interactions with different types of drugs. In 2005, Hjerde et al. [12] approached D2’s interactions with typical (haloperidol and loxapine) and atypical (clozapine and melperone) antipsychotic drugs. The study concluded that D2DR has higher nonbonded interactions with typical drugs, with a lower displacement induced in TMH5. Other studies have used MD simulations and quantum mechanics (QM) to describe D2DR’s interaction with distinctive ligands. For example, Andujar et al. [13] focused on whether tetrahydroisoquinolines act as dopaminergic ligands, while computational modeling techniques were used to determine the mechanistic role of helix 8 in GPCRs (G protein-coupled receptors) found at the surface of cells that detect molecules outside the cell and activate cellular responses [14]) and dopamine D2 receptor’s interaction with the GIPC1–PDZ domain [15]. GPC receptors present positively charged residues on the cytoplasmic matrix of membranes (Figure 2) and are mainly found under α-helical conformations. D2DR’s most favorable interactions in transmembrane models were observed around arginine and lysine residues. At the membrane surface, these residues are known to form abundant positively charged rings that are complementary to electronegatively charged surfaces [14].

The present article focuses on the potential binding positions in which the three previously discussed synthetic compounds (RACL, FLB, and SCH) used in medical imaging (PET) investigations would most likely interact with the D2 dopamine receptor. The motivation behind this study relies on the highly debated aspects within the literature related to the three widely used radioligands: their specific radioactivity (here we were interested in their binding sites), their uptake mechanisms (structural and dynamical properties), and—maybe the most debated and important matter in clinical trials—their selectivity. For the latter aspect, the chemical and biochemical interactions between the ligand’s molecular structure and different parts/subparts of the living organism can be accurately addressed only through experimental approaches. However, the biophysical characterization of these radioligands provides additional insights into the interaction mechanisms between the vehicle molecules and their targets.

Both sets of the resulting docked structures, one using a blind docking technique (for the top section of D2DR) and another using a specific docking approach with a particular grid search method (for the bottom part of the receptor), were subjected to further classical MD simulations for structural and dynamical characterization.

## 2. Results

### 2.1. Docking Analysis

From the blind docking approach considered for the ligand binding modes at the top of the receptor (Table 1), a total of 48 clusters for FLB, 43 resulting clusters for RACL and 33 clusters for SCH were investigated. For the same position, the FLB ligand resulted in 28 docked clusters, RACL had 19 docked positions, and SCH presented 21 clusters of potential interest. The resulting clusters docked at the external parts of D2DR presented lower docking scores (and much lower Δ*G* absolute values) and were not considered for further investigations.

For the bottom part of the receptor (Table 2), we considered a specific grid search method delineated by the following parameters: the center of the box was set at 45 × 44 × 69 Å with 68 points in the x-dimension, 66 in the y-dimension, and 50 in the z-dimension. From the total number of 45 and 42 resulting clusters for the FLB ligand and RACL ligand, respectively, 31 clusters were docked inside D2DR’s for the FLB ligand, while for RACL we obtained a lower number of 27 clusters. However, only 13 docked clusters were found for the SCH ligand at D2DR’s bottom position, and this number was registered as the lowest among all ligands.

The main D2DR residues involved in the interactions (within 2.5–4.1 Å) with the FLB ligand (docked at the bottom part of D2DR) are LYS-191, ILE-252, GLU-189, ALA-30, -101, and -192 (Figure S1).

The residues involved in the RACL–D2DR interactions (at 2.1–3.4 Å distances) were identified as PHE (210 and 76), ILE-146, SER-230, and GLU-61 (for RACL docked at D2DR’s top pocket). At the bottom level of the receptor, the RACL ligand presented similar binding affinities (within 2.1–3.6 Å distances) around the LEU-31, THR (33 and 35), ASN-36, ALA-101, and PHE-254 residues (Figure S2). 

The most favorable SCH-receptor interacting sites (docked at D2DR’s top pocket) were described by distances ranging between 2.6 and 3.6 Å near the TRP-234 and THR-233 residues (Figure S3). For the same docked ligand at the bottom part of the receptor, lower binding affinities were related to the ASN-251, LEU-31, ARG-98, and THR-35 residues (at 2.0–3.5 Å distances between the ligand and the receptor).

The most frequently employed residues in D2DR–ligand interactions were ASN-36, ALA, THR (both 33 and 35), LEU-31, and ARG-98 for the docked positions at the bottom part of the receptor. On the other hand, the results showed that for the ligands docked at the top of D2DR, fewer residues (SER-230, GLU-61, and ILE-146) were involved.

### 2.2. Solvent Accessible Surface Area

**Figure 3:**
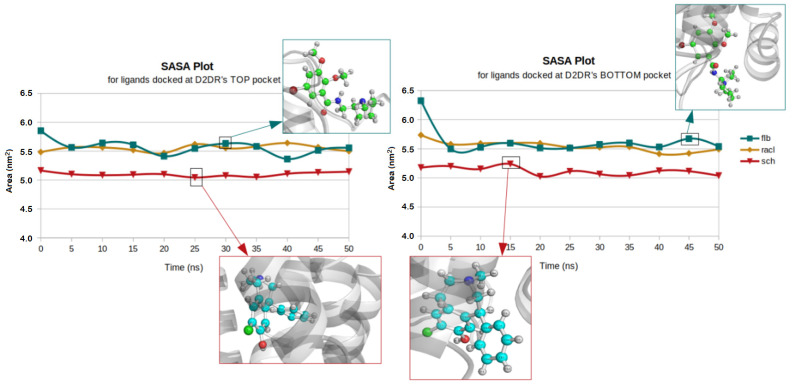
SASA profiles for ligands docked at D2DR’s top (**left**) and bottom (**right**) positions.

The hydrophilic and hydrophobic profiles (Figure 3) showed lower values for the ligands docked at the top part of the receptor when compared to the bottom docked position, indicating that all the ligands remained inside the docked position of D2DR. The highest SASA average value of 5.63 nm^2^ was noted for the FLB ligand docked at the bottom part of the receptor.

The aliphatic chain of the FLB ligand also showed great dynamic fluctuation rates due to D2DR’s secondary structure components at its lower part, where random coils and turns are more abundant. The maximum measured SASA value noted for the same ligand and in the same docked position was 6.32 nm^2^.

The highest retention from solvent exposure was also measured for the SCH ligand, which was docked at the top part of D2DR with an average value of 5.10 nm^2^. For the same ligand, in the opposite docked position, we observed a short and spontaneous behavior at approximately 15 ns when the SCH aromatic C6 ring became highly exposed to solvent molecules. In contrast, a well-balanced behavior between the two D2DR docked positions was identified for the RACL ligand, having similar SASA values. At D2DR’s top pocket, RACL showed an average value of 5.55 nm^2^, while for the bottom docked position its SASA value was 5.54 nm^2^.

Another aspect that must be considered for the solvent accessibility profiles of ligands is their molecular geometry. The ligand with a shorter chain and, consequently, a lower number of atoms (SCH consists of 38 atoms) presented diminished solvent exposure profiles for both receptor extremities.

On the other hand, RACL (with 42 atoms) and FLB (with 45 atoms) were more susceptible to hydrophilic behaviors because their aliphatic chains were larger and prone to interact with the solvent molecules (FLB > RACL > SCH). Except for the situation previously mentioned (related to SCH), for all three ligands the phenyl groups (cyclic C_6_H_5_- groups) maintained their hydrophobic chemical motifs during the entire simulation time of 50 ns.

### 2.3. Radius of Gyration

**Figure 4:**
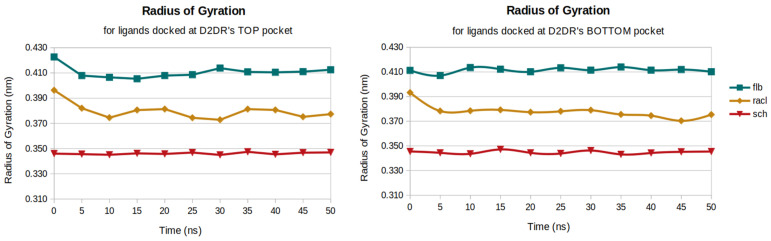
Radius of gyration profiles for the two sets of ligands.

The receptor Rg values were not considered in the present study because the secondary structure analysis of D2DR presented no significant variations and the alpha-helical content was the most abundant structural element. The gyration profiles between the top and bottom docked positions are very similar, which denotes no significant structural changes for the ligands when different interacting D2DR sites are considered (Figure 4). For both D2DR docked positions, the FLB ligand showed the highest average gyration radius of 0.41 nm.

The lowest Rg average value of 0.34 nm was observed for the SCH ligand docked on both D2DR’s interacting sites, although larger changes in the ligands’ compactness profiles during the simulations were obtained for the RACL ligand docked on both pockets of the receptor. These changes occurred in the first 10 ns of simulation time and converged to a gyration average value of 0.38 nm.

### 2.4. Root-Mean-Square Fluctuations

The RMSF results indicate that the highest atomic fluctuations with an average RMSF value of 0.15 nm correspond to the RACL ligand from the bottom part of the receptor, while the lowest fluctuations of 0.06 nm were seen for the SCH ligand situated in the same position. At the top part of D2DR, the FLB and RACL ligands showed the same average RMSF value of 0.12 nm.

**Figure 5:**
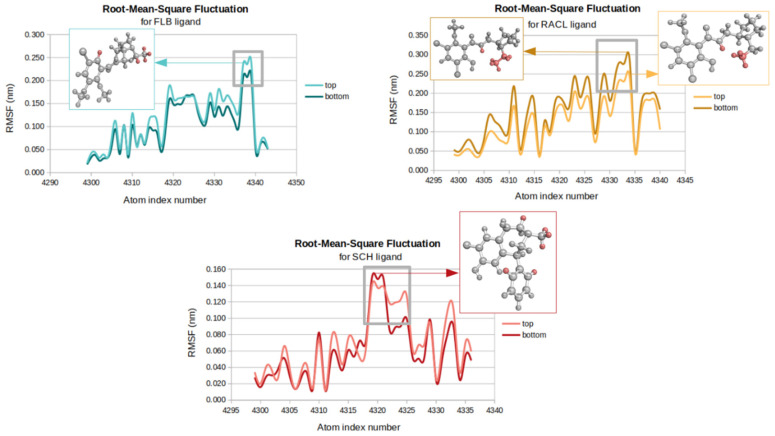
RMSF plots of D2DR’s docked ligands.

The lower fluctuations of the SCH ligand (Figure 5) are consistent with the lower gyration values of 0.34 nm and with the high hydrophobic profiles described by the lowest SASA values for both D2DR docked positions. In the same manner, a strong correlation between RACL’s increased flexibility and its gyration behavior can be observed when its gyration profiles are considered.

The lowest average values related to the SCH ligand docked on both D2DR docking positions were due to high fluctuations of just six individual H atoms, three of which were involved in the reduced number of degrees of freedom corresponding to the short ligand’s aliphatic chain.

In contrast, the higher fluctuation rates observed for the RACL and FLB ligands located at D2DR’s bottom part are characterized by strong vibrations of the CH_3_-CH_2_- atoms from their straight-chain alkyl groups, while for the same ligands docked at the top of the receptor the most abundant motions correspond to the methyl (CH_3_-) groups from their aliphatic terminal chain.

The RMSF measurements are in good agreement with the obtained SASA profiles and the gyration behavior of all the ligands. For the elevated atomic fluctuations of the RACL and FLB ligands, we obtained the highest solvent exposure areas on both docked positions of the receptor, which, therefore, increased the gyration values.

### 2.5. Root-Mean-Square Deviations

Overall, the ligands docked at the D2DR top position showed similar fluctuation rates when compared to the ligands from the bottom part of the receptor. The most unstable compound was the FLB ligand situated on both extremities of D2DR—with RMSD average values of 0.20 nm for the top docked position and 0.19 nm for the bottom part. Another high RMSD of 0.18 nm was observed for the RACL ligand situated at the bottom part of D2DR.

**Figure 6:**
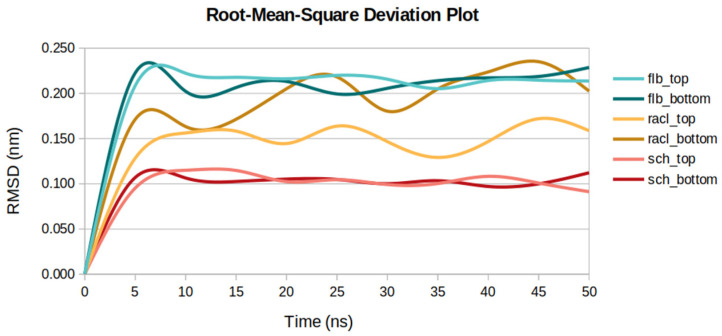
RMSD values for the three ligands docked at the top and bottom parts of D2DR.

If we consider the differences between the ligands docked at the superior and inferior parts of the receptor, the SCH ligand showed the lowest RMSD of 0.09 nm for both D2DR positions. However, for the RACL ligand, the RMSD profile (Figure 6) shows slightly higher atomic deviations for D2DR’s bottom part of 0.18 nm when compared to the top docked position where the average RMSD value was approximately 0.14 nm.

Moreover, the RMSD profiles for the SCH ligand (with the lowest average value of 0.09 nm) docked on both parts of the receptor are in good agreement with the ligand’s Rg minimum value of 0.34 nm, and its lowest RMSF profiles of 0.07 nm for the top D2DR docked position and 0.06 nm for the bottom D2DR position.

### 2.6. Root-Mean-Square Deviation of Atom Distances

The root-mean-square of the differences in atom-pair distance calculations have the advantage of not using the least-square fitting of the structures to the reference ones, as in the case of a standard RMSD analysis. The average atom-pair distances were extracted from multiple trajectories for each ligand docked on both docked positions. These measurements were used for further structural analysis of the ligands in terms of their mobility and dynamic stability.

**Figure 7:**
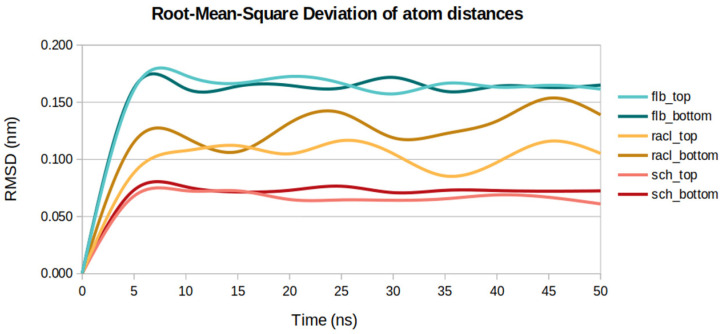
Atom-pair distances for the three ligands docked at the top and bottom parts of D2DR.

In agreement with high RMSF profiles for the FLB ligand, the maximum atom-pair average distance of 0.15 nm was obtained for the same ligand docked on both receptor positions. The average atom-pair distances (Figure 7) slightly increased for the ligands situated at the bottom part of the receptor when compared to those docked at the top part of D2DR. For example, the RACL ligand’s average values increased from 0.094 nm (docked at D2DR’s top position) to 0.116 nm (docked at D2DR’s bottom position).

The lowest atom-pair distances among all the ligands for both D2DR docked positions were observed for the SCH ligand, with an average atom distance value of 0.06 nm for the top part of the receptor and 0.07 nm for the bottom part of the receptor. Additionally, the atom-pair distance values for SCH are strongly correlated to the ligand’s RMSF average value of 0.068 nm and its lower gyration profile at both receptor positions of approximately 0.34 nm.

As expected, the RACL atom-pair distances corresponded to the ligand’s RMSD variations (0.04 nm between the top and bottom positions). Moreover, a difference of 0.03 nm was seen when the top docked position was compared to the bottom one—the same difference as observed for RMSF measurements (0.12 nm for RACL docked on the top part of the receptor and 0.15 nm for RACL situated at D2DR’s bottom part).

### 2.7. Angular Distributions

The average value of a group of angles as a function of time is a key measurement for the structural deformations or bending profile analysis. The ligands’ flexibility rates were obtained using the incorporated *angle* tool from the Gromacs package.

**Figure 8:**
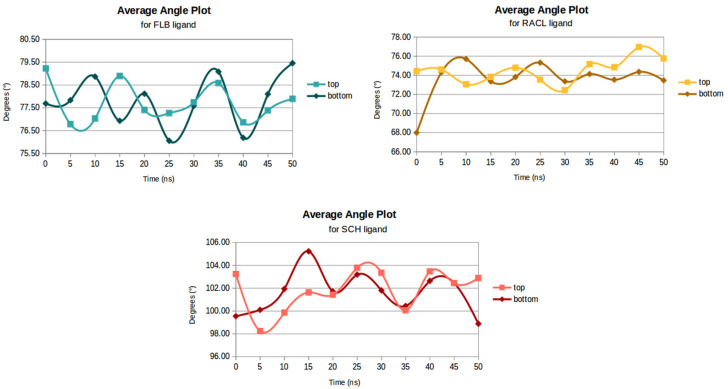
Average angle plots for each set of ligands.

Our results (Figure 8) showed smaller angles for the RACL ligand docked at the bottom part of the receptor when compared to the same ligand docked at D2DR’s top position. The average angle decreased from 74.49° (for a ligand docked at D2DR’s top position) to 73.57° (for D2DR’s bottom docked position). For the FLB and SCH ligands, the values were almost the same when different parts of the receptor were compared. The largest angles of 101.85° and 101.63° were obtained for the SCH ligand docked at the receptor’s top part and at the receptor’s tail, respectively.

Another important finding is that all the angles, analyzed as a function of time, showed significant decreases between the FLB and RACL ligands docked at the top and at the bottom of D2DR. For example, considering only the top docked positions, FLB presented an average angle value of almost 78°, while for the same docked spot, RACL had an average angle of 74.5°. A related behavior from 77.81° (for FLB ligand) and 73.57° (for RACL ligand) was also seen for the D2DR bottom docked position.

From an intrinsic dynamical point of view, the FLB and SCH ligands presented transient stability with higher fluctuations between the 5 ns and 35 ns timescales. The RACL ligand had a starting average angle of 67.98°, and, after 20 ns, its angles significantly increased to 75.31°. However, for the SCH ligand, despite the large values, the angles were relatively stable, thereby presenting the smallest changes during the 50 ns MD run.

Moreover, the most significant effects on the angle distributions were seen for the FLB and RACL ligands docked at the bottom part of the receptor, where their angles ranged between 76–79° (for FLB) and 68–75° (for RACL). At the bottom level of D2DR, these two ligands outlined the intention of leaving the docked pocket indicated by substantial conformational changes (the second C6 rings for each ligand slightly shifted out of D2DR’s bottom pocket).

### 2.8. Principal Component Analysis

The dominant modes of the ligands’ collective motions were extracted from the final corrected trajectories using the PCA tool. The overall rotational and translational motions of each ligand were eliminated by imposing a least-squares fit superimposition onto the ligand’s reference structure and by a translation to their average geometrical center. PCA was carried out on all atom–ligand models, and, for the last 30 ns (20,000–50,000 ps), using 300 frames per run.

Most of the motions (approximately 80% of the total mobility) per ligand in each receptor’s docked position are described by the eight principal components illustrated in Figure S4.

**Figure 9:**
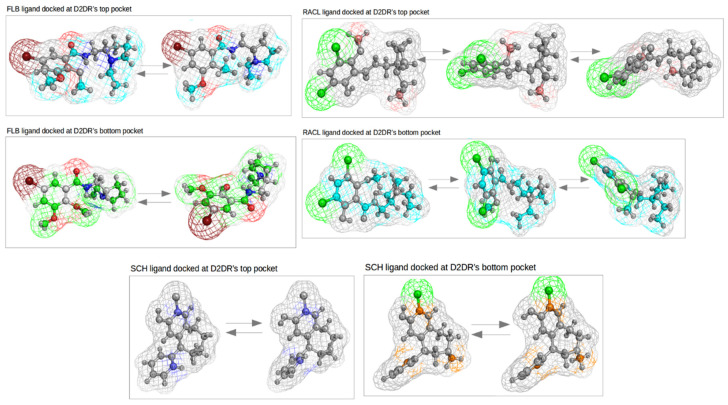
The most prominent motions extracted from PCA measurements for the three sets of ligands.

The most prominent modes observed for the FLB ligand on both docked positions are characterized by the CH_3_, C-Br, and -N-CH_2_-CH_3_ aliphatic group movements. For the ligand docked at D2DR’s top pocket, the rotatable bonds illustrated in Figure 9 promote drastic conformational changes of the entire structure, and consequently favor its translational and torsional motions. The ethylamine (CH_3_-CH_2_-N-) component has, in most cases, a rotary type of motion.

Another set of independent motions that triggered RACL’s curved-twisted shape and, as a result, presented the highest eigenvector fluctuations are the peripheral -CH3 and -C-Cl groups drawn in Figure 9 (at the top and to the right). For the same ligand docked at D2DR’s top pocket, its conformational changes were also outlined by bending motions, but implied fewer methyl groups and, consequently, lower RMS fluctuations.

An interesting dynamic behavior, but in good agreement with RMSD and the gyration profiles, is related to the SCH ligand, which shows very low flexibility. The first two principal components for the ligand docked at D2DR’s top and bottom parts are almost identical. For the top docked position, only two C atoms and several peripheral H atoms were involved in the ligand’s collective motions. Slightly higher fluctuations were observed for the SCH ligand docked at the bottom of the receptor, where its principal components involved the rotatory peripheral C-Cl and -CH3 group movements.

### 2.9. Total Interaction Energies

The total interaction energies were calculated using two components: Coulombic short-range interactions and Lennard–Jones short-range potentials. The results (Table 3 and Table 4) show that the interaction energies for the D2DR–RACL ligand docked at the receptor’s top pocket presented the highest average value of −150.04 kJ/mol and that the SCH ligand presented acomparable total energy of −147.59 kJ/mol. Surprisingly, for the ligands docked at the bottom part of D2DR, the highest interaction energy of −164.22 kJ/mol was observed for the SCH ligand. Moreover, an interesting energetic behavior was seen for the SCH and RACL docked ligands, where their energy patterns were very similar considering their different docked positions. For example, an energy value of −147 kJ/mol was observed for the SCH ligand docked at D2DR’s top position, whereas the same interaction energy was observed for the RACL ligand docked at D2DR’s bottom docked position. This leads to the approximation that the strength of interactions between RACL and D2DR at its bottom part is similar to the strength of interactions between SCH and the receptor at its top part.

Lower total interaction energies of −135.50 kJ/mol and −106.13 kJ/mol were observed for the FLB molecule docked at D2DR’s top pocket and at its bottom position, respectively. Overall, the ligands situated at the top part of D2DR converged to higher energy patterns in comparison to the ligands situated at the bottom part of the receptor. On the other hand, the SCH ligand docked at the bottom part of D2DR showed the highest interaction energy value. Another interesting result is that for the RACL and SCH ligands docked on both receptor pockets, their opposite structural behaviors were correlated with the highest energy patterns among all ligands.

Although the macromolecules’ configuration depends on the known amino acid sequences and their building blocks’ assembly, the ligands describing small and highly flexible molecules may exhibit distinctive shapes and topologies.

As previously mentioned in Section 2.8, the ligand’s configurations play a crucial role in ligand binding patterns. As a result, at the bottom docked position of the receptor, the SCH ligand presented a constant structural behavior and, therefore, was correlated with a higher binding affinity. However, for the same docked position but for the other two ligands, their partially distorted configurations due to the high flexibilities of their aliphatic chains were correlated with lower L-J-SR interactions.

In contrast, for the receptor’s top docked position, the strength of interactions between the RACL ligand and D2DR is slightly higher but similar to the one corresponding between SCH and the receptor. In this context, the binding kinetics and free energy calculations for a more accurate description of the thermodynamic contributions, particularly for the top docked position of D2DR, will be considered for the near-future investigations. 

Our interest in the SCH-23390 ligand was mainly related to the hypothesis that even if it is a D1 subunit antagonist, the compound might also have minimal effects on the D2 subunit type of receptor [10,16], and, to our knowledge, there are no other previous theoretical studies focused on SCH-D2 interacting complexes.

### 2.10. Available Experimental Data

Considering the top docked position of D2DR, our results are in good agreement with the experimental data focused on [^11^C]RACL and [^11^C]FLB 457 radioligand binding affinities, although these studies qualified D2DR quantification in extrastriatal regions as being challenging because, even if specific binding is detectable, this is not necessarily an indicator of adequate precision or validity of extrastriatal measurements [17]. For example, the same study revealed a medium affinity of [^11^C]RACL in high-density striatum levels and low occupancy of the same radioligand in extrastriatal regions, with low to moderate uptake of both [^11^C]RACL and [^11^C]FLB [17]. In contrast, in low-density extrastriatal regions, other studies showed high affinity of [^11^C]FLB for D2DR [18,19,20]. For the same compound, but in high-density regions of the striatum, the radioligand was almost impossible to quantify because it did not reach the equilibrium state within a feasible scanning time period [21,22].

The test–retest data demonstrated that the [^11^C]RACL ligand, over time (post 29 months), presented significantly reduced levels in the frontal and temporal cortex and in the striatal areas [23]. In contrast, from the same type of measurements, the [^11^C]RACL radioligand presented increased binding potential indicators in the striatum, thalamus, and temporal cortex [24,25,26,27], therefore suggesting that the radioligand is not actually suitable for D2DR quantification in extrastriatal regions [27] and that the question regarding its reliability remains to be elucidated [17]. On the other hand, the [^11^C]FLB 457 ligand was correlated with highly specific binding potentials in extrastriatal areas [28]. In addition, other experimental studies concluded that the [^11^C]FLB radioligand is able to quantify low-density dopamine receptors as well [21,29], and is generally considered a suitable radiotracer for the cortical brain, thalamus, and other extrastriatal D2DR-binding regions [19,20,30,31,32].

It must be kept in mind that the relative uptake of these radioligands in different cerebral regions does not only depend on the receptor densities, while the regional signals also consist of free ligand in tissues and nonspecific contributions [33]. Moreover, the intravascular activities and the tracer’s post-injection timescale have a direct impact on the specificity of the bindings within particular brain areas [33]. Finally, for certain disorders, the agonist treatment might also affect the receptor bindings by suppressing the hypersensitivity processes expected in that particular disorder (e.g., Parkinson’s disease) [33].

In addition, the present study focuses strictly on the biophysical description of the interaction mechanism between the three ligands and one of the two major dopamine receptor subtypes (D2 subtype), including their dynamical and structural properties. The results show, in agreement with the experimental data [34], that the ^11^C-RACL ligand docked at the top part of the receptor is a reliable radiotracer for D2DR quantification. With slightly different energetic patterns [17], the ^11^C-FLB ligand can also be considered as a promising candidate for D2DR quantification [18,19,20,28], especially because, from a structural point of view, it behaves similarly to the RACL ligand. However, the ^11^C-SCH ligand presents a constant structural and dynamical behavior during the MD productions, with a potential affinity not only for D1 receptors but also for the D2 subtypes [10,16].

### 2.11. ONIOM (QM:QM’) Calculations

**Figure 10:**
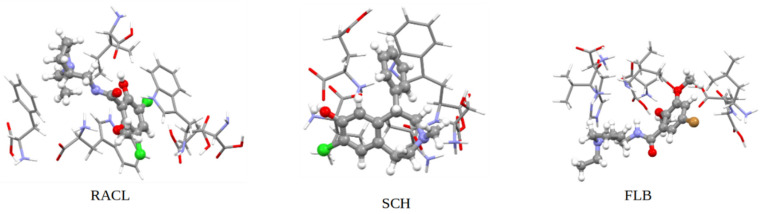
Optimized geometries of the investigated complexes at D2DR’s top docked position at the ONIOM (ωB97XD/6–311+G (d,p):ωB97XD/3–21G) level of theory.

The QM:QM’ ONIOM method [35] was used for the geometry optimizations and frequency calculations of the complexes formed by the radiopharmaceutical ligands and protein residues located within 4 Å of the ligand (see Figure 10). For these calculations, the range-separated and dispersion-corrected ωB97XD hybrid functional [36] was used in combination with the 6–311+G(d,p) basis set, as implemented in the Gaussian program [37]. Here, we used a two-layer ONIOM scheme, where the real system includes all the atoms and is calculated at the ωB97XD/3–21G level of theory (QM’), while the model system, calculated at the ωB97XD/6–311+G(d,p) level of theory (QM), contains only the radiopharmaceutical ligand.

The total energy of the system is obtained using the formula [38]:

(1) $$E_{\mathrm{ONIOM}}=E_{\mathrm{model}}^{\mathrm{QM}}+E_{\mathrm{real}}^{\mathrm{QM}’}-E_{\mathrm{model}}^{\mathrm{QM}’}$$

To define the convergence of the molecular geometries, we used tight criteria, while, for the electronic density, very tight criteria were applied. An ultrafine grid was used for the numerical integration of the electronic density. The lack of imaginary frequencies confirmed that for all the investigated complexes, the optimized geometries correspond to minima on the potential energy surfaces.

The interaction energies between the radioligands and the protein residues were defined as in ref. [39]:

(2) $${\Delta E}_{\mathrm{int}}=E_{\mathrm{complex}}-E_{\mathrm{ligand}}-E_{\mathrm{residues}}$$

To calculate the basis set superposition error correction (BSSE), the standard counterpoise method [40] was used and the necessary single point calculations were performed on the ONIOM (ωB97XD/6–311+G(d,p):ωB97XD/3–21G) optimized geometries using the same basis set, 6–311+G(d,p), for all the atoms in the complexes. For these calculations, the systems were divided into two fragments: one fragment was the ligand and the second was formed by the set of residues from each complex.

The ONIOM (QM:QM’) calculations were considered only for the top-docked position of D2DR, because when it is bound to a membrane its top pocket would be the most favorable docking site. As reported in Table 3, the total interaction energy between D2DR and the FLB ligand docked at the top position was the lowest (−135.50 kJ/mol) among all the ligands. In the same manner, the ONIOM calculations predict the lowest interaction energy for the same ligand with a complexation energy of −164.60 kJ/mol.

In good agreement with the total interaction energies from MD trajectories, the SCH ligand is situated between the maximum and minimum complexation energy values, implying that for a more detailed electronic description, the ligand’s docked position has little influence on its conformational adaption inside the receptor.

As expected, and already confirmed by our MD reported data, the RACL ligand docked at D2DR’s top pocket presented the maximum complexation energy among all ligands. For the RACL–D2DR complex, the complexation energy is −255 kJ/mol.

These obtained ONIOM energies can be easily correlated with high fluctuations of the ligand’s RMSD and RMSF profiles while remaining inside the docked spot during the entire MD production time. The complexation energy gap between the RACL and SCH ligands treated at the QM:QM’ level was just −25.55 kJ/mol. An even smaller (−2.45 kJ/mol) energy gap between the two ligands was noted for the total interaction energies obtained from MD productions. The latter finding indicates that, regardless of the chosen calculation method, for the top docked D2DR position, the SCH compound (with a complexation energy of −229.45 kJ/mol) represents the second most suitable binding ligand.

## 3. Materials and Methods

The receptor (D2 subunit) coordinates were retrieved from the Protein Data Bank (PDB Code 6CM4) database [3]. Using the PyMOL [41] visualization program and UCSF Chimera [42] software, prior to MD simulation, a dock-prep of the receptor and ligands was considered where partial charges and hydrogen atoms were added. The topology files for all the complexes (receptor and ligands) were generated using the GROningen MAchine for Chemical Simulations (GROMACS) package [43]. The CHARMM36 force field [44] was used for ions, water molecules, and D2 receptor parameters. The ligand parameters were generated using the CGenFF (CHARMM General Force Field) server [44].

The preferential binding positions were defined using a molecular docking approach [45,46]. The docking calculations were carried out on the Swiss Dock server [47], from which the obtained clusters were ranked according to their full-fitness (FF) scoring function. The results showed two preferential positions (with higher FF score absolute values) at the receptor level (top and bottom pockets) for all the ligands, totaling six docked positions considered for further investigations.

The receptor–ligand docked complexes were then solvated with a TIP3P water model [48]. To remove any potential steric clashes, the systems were minimized in a set of 50,000 steps using the steepest descent minimization algorithm. Moreover, chloride ions were added to neutralize each system.

The receptor–ligand complexes were then equilibrated for 10 ns in an NVT ensemble, where the temperature was regulated at 310 K via a modified Berendsen thermostat [49]. The temperature coupling was assigned using two separate groups (D2DR ligand with water ions) using a time constant of 0.1 ps. The last frame from the NVT ensemble was used for the following NPT equilibration for 10 ns in isotropic pressure couple type with a time constant of 1.0 ps and a reference pressure of 1 bar. The ligands were slightly restrained during both, the NVT and NPT ensembles.

For each simulation, H-bond holonomically applied constraints based on the LINCS algorithm [50] were used, and the particle mesh Ewald (PME) [50] calculation method was considered for long-range electrostatic description. Furthermore, the Coulombic and van der Waals interactions were described using a cut-off distance of 12 Å.

Multiple MD run productions were performed with periodic boundary conditions for 50 ns using a time step of 2 fs in the NPT ensemble with no additional restrictions. The trajectory analysis was performed with the implemented tools from the Gromacs package.

## 4. Conclusions

Our docking results predicted that the FLB and RACL ligands tagged with ^11^C isotopes present higher binding affinities at the top part of the receptor. The absolute interaction energies at this level were 147.78 kJ/mol for RACL and 106.13 kJ/mol for the FLB ligand. The highest absolute interaction energy for the bottom part of the receptor was observed for the SCH ligand, while, for the top part of D2DR, the highest interaction patterns were observed for the RACL ligand. Additionally, from our predicted docked structures, the highest docking scores were seen for the RACL ligand at D2DR’s top position and the FLB ligand for the opposite docked pocket. While the FLB ligand manifests the tendency to emerge out of the receptor’s bottom pocket, the SCH and RACL ligands showed greater binding affinities for both receptor pockets while remaining inside of D2DR. As a consequence, the maximum solvent exposure area of 5.63 nm^2^ was observed for the FLB ligand docked at D2DR’s bottom position.

The Rg profiles for all the ligands suggested a constant behavior in the ligand compactness levels, especially for the FLB and RACL ligands, which had the same gyration average values on both D2DR docked positions. For the SCH ligand, the Rg average value slightly decreased when the two opposite docked positions were considered. The highest atomic RMS deviations were observed for the FLB ligand situated at the top of the receptor. Likewise, the highest interatomic distances were correlated with the same ligand but considered at both D2DR positions.

For both extremities of D2DR, all the ligands showed a similar angular distribution behavior. The most significant effects on the angle distributions were seen for the FLB and RACL ligands docked at the bottom part of D2DR, although the SCH ligand presented the largest angle value distributions, but without significant structural changes. The most prominent motions extracted from the PCA measurements for the RACL ligand docked at D2DR’s bottom position were triggered by the peripheral aliphatic -CH3 groups and by the -C-Cl aromatic ring groups. This type of motion led to a curved-twisted shape of the FLB and RACL ligands, which is in good agreement with their high atomic RMS fluctuations.

Moreover, the QM calculations confirm this preferential top docked position of D2DR for all the ligands and identifies the RACL ligand presenting the highest complexation energy, with the SCH compound being the second most preferred ligand.

Thus, according to our results, the RACL ligand docked at the top pocket of D2DR and the SCH ligand considered at the bottom part of the receptor are clearly the most efficient ligands for the modeled receptor–ligand interacting complex herein. As perspectives, we consider further MD investigations with extensive production time and free energy calculations of the same docked complexes at the receptor’s top docked position with the D2DR structure embedded in a phospholipid bilayer membrane.

## Figures and Tables

**Table 1:** Molecular docking results for D2DR-ligand top complexes.

FLB		RACL		SCH	
Full-Fitness Score	Δ*G* (kcal/mol)	Full-Fitness Score	Δ*G* (kcal/mol)	Full-Fitness Score	Δ*G* (kcal/mol)
−1168.39	−7.954	−1171.87	−7.616	−1134.33	−7.126

**Table 2:** Molecular docking results for D2DR-ligand bottom complexes.

FLB		RACL		SCH	
Full-Fitness Score	Δ*G* (kcal/mol)	Full-Fitness Score	Δ*G* (kcal/mol)	Full-Fitness Score	Δ*G* (kcal/mol)
−1157.05	−7.44	−1156.43	−6.899	−1103.53	−4.929

**Table 3:** Total interaction energies (calculated as the sum between Coulombic and Lennard-Jones interactions) for the three ligands docked at D2DR’s top position.

FLB		RACL		SCH	
Coul-SR (kJ/mol)	L-J-SR (kJ/mol)	Coul-SR (kJ/mol)	L-J-SR (kJ/mol)	Coul-SR (kJ/mol)	L-J-SR (kJ/mol)
−11.09	−124.40	−22.64	−127.40	−30.33	−117.26
Total Energy: −135.50 kJ/mol		Total Energy: −150.04 kJ/mol		Total Energy: −147.59 kJ/mol	

**Table 4:** Total interaction energies (calculated as the sum between Coulombic and Lennard-Jones interactions) for the three ligands docked at D2DR’s bottom position.

FLB		RACL		SCH	
Coul-SR (kJ/mol)	L-J-SR (kJ/mol)	Coul-SR (kJ/mol)	L-J-SR (kJ/mol)	Coul-SR (kJ/mol)	L-J-SR (kJ/mol)
−10.01	−96.12	−24.84	−122.94	−26.15	−138.07
Total Energy: −106.13 kJ/mol		Total Energy: −147.78 kJ/mol		Total Energy: −164.22 kJ/mol	

## Data Availability

All data generated or analyzed during this study are included in this published article.

## Supplementary Materials

The following supporting information can be downloaded at: https://www.mdpi.com/article/10.3390/ijms23042005/s1.
