# Supplementary material for: All-Atom Molecular Dynamics Investigations on the Interactions between D2 Subunit Dopamine Receptors and Three 11C-Labeled Radiopharmaceutical Ligands
Source: Int J Mol Sci. 2022 Feb 11;23(4):2005. doi: 10.3390/ijms23042005 (PMC8880249; doi:10.3390/ijms23042005)
Supplement: Supplementary file 1 [file ijms-23-02005-s001.zip › ijms-1572495-supplementary.pdf]

International Journal of
Molecular Sciences

MDPI

Supplementary Information

Docking Analysis

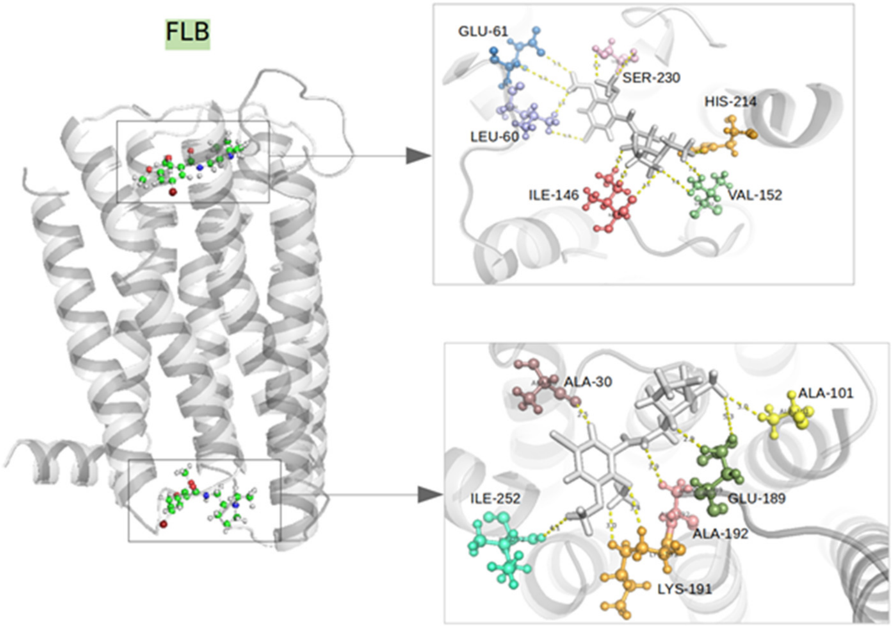

Figure S1. Interacting docked residues of D2DR against FLB ligand.

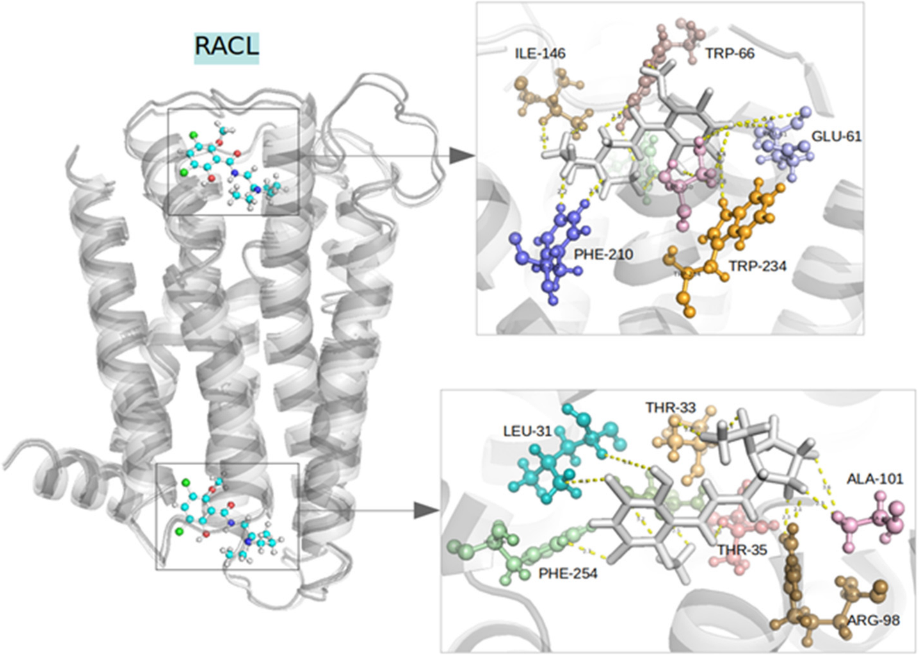

Figure S2. Interacting docked residues of D2DR against RACL ligand.

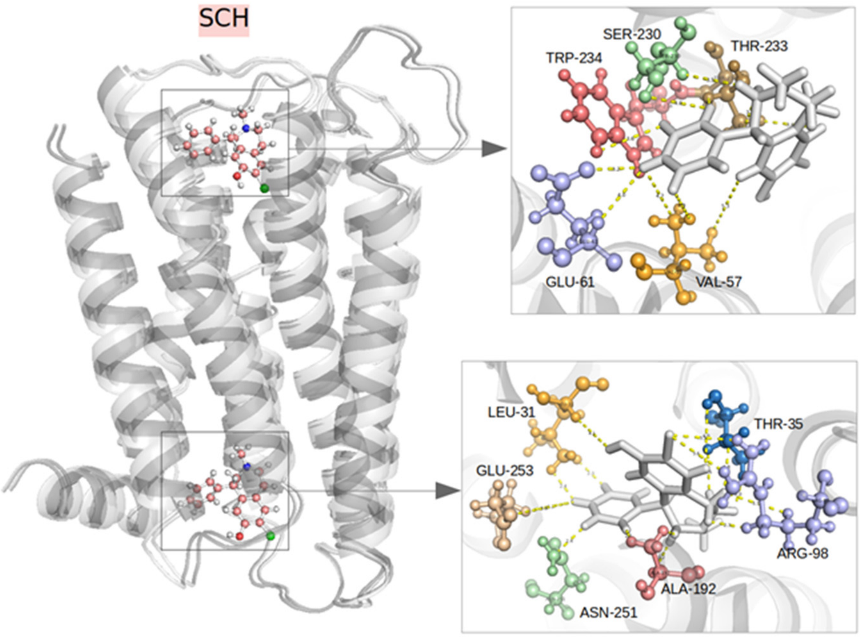

Figure S3. Interacting docked residues of D2DR against SCH ligand.

2.8. Principal Component Analysis

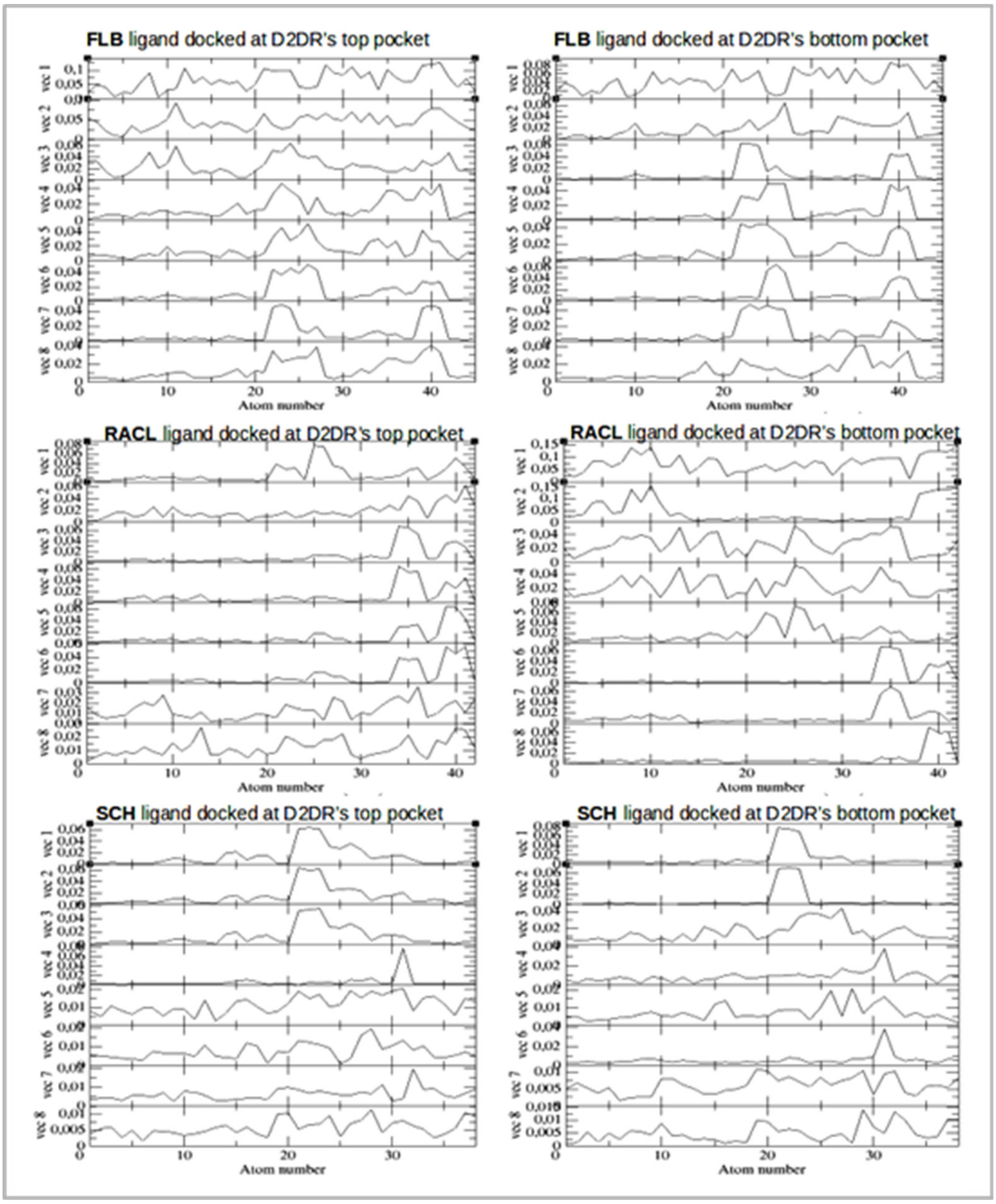

Figure S4. RMS fluctuations (in nm) derived from PCA analysis of the output trajectories of all ligand atoms for the first 8 eigenvectors.

Analysis of Variance (ANOVA)

Using slightly different but equally possible starting configurations of the docked complexes, and on both D2DR pockets, we performed 8 replicas of 50 ns MD productions. In addition, each replica was treated separately in terms of solvation, energy minimization, and NVT/NPT equilibrations.

Each blue box represents one replica of 50 ns production period.

As a result, the analysis of variance show no run-to-run statistically significant differences (see figures below). This finding implies that the ligands presented similar

conformations (the samples come from the same distribution) and dynamic behavior along the trajectories, on each docked position and within each sample.

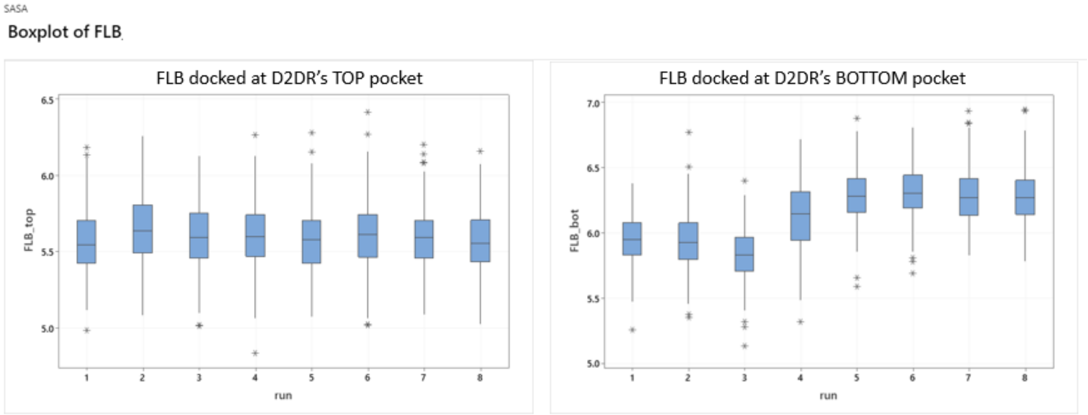

Figure S5. Boxplots of the solvent exposure area (SASA) from the two FLB simulation sets (top and bottom).

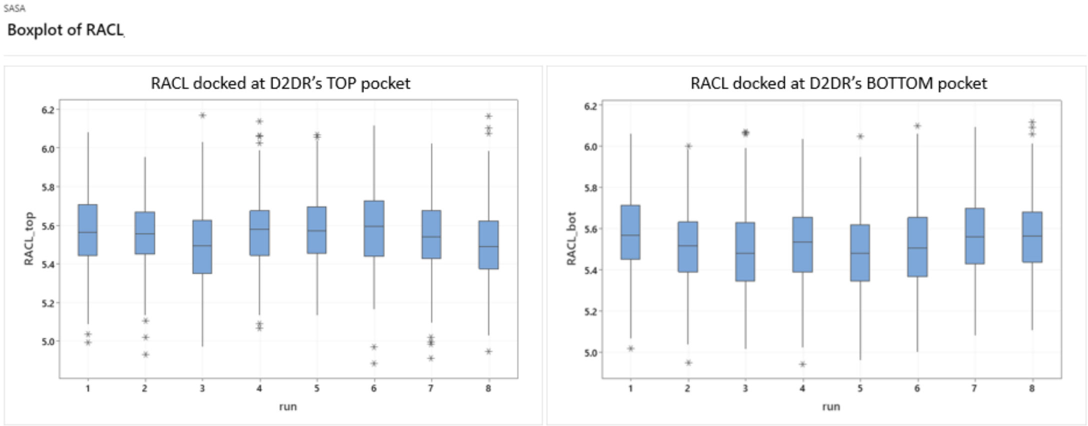

Figure S6. Boxplots of the solvent exposure area (SASA) from the two RACL simulation sets (top and bottom).

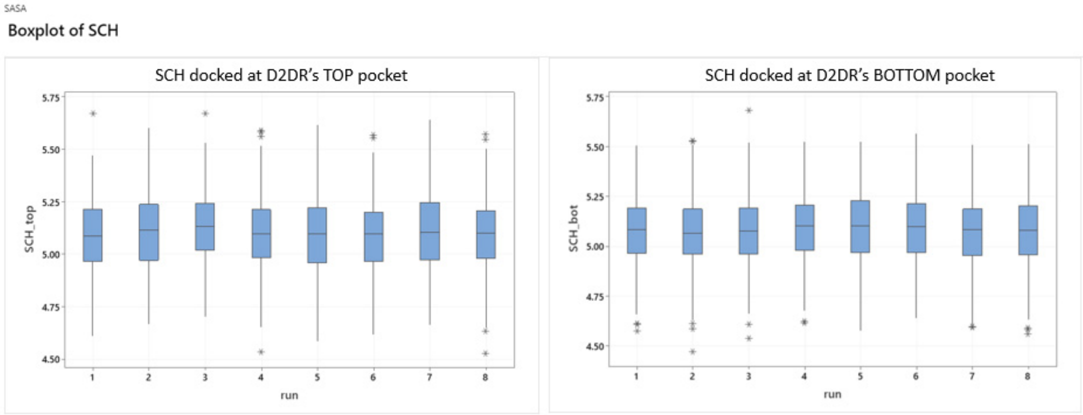

Figure S7. Boxplots of the solvent exposure area (SASA) from the two SCH simulation sets (top and bottom).

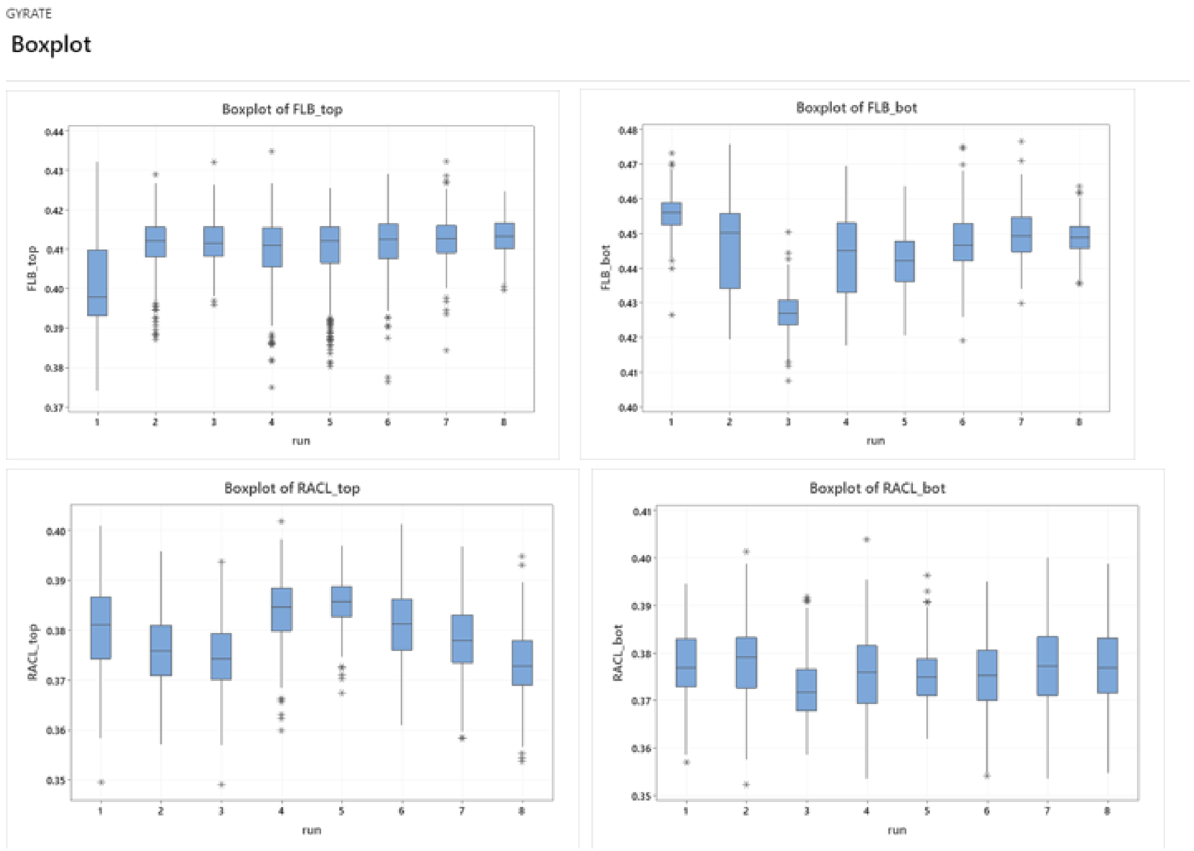

Figure S8. Boxplots of the gyration radius (Rg) from: the two FLB simulation sets (top-left and top-right), and the two RACL simulation sets (bottom-left and bottom-right).

The FLB and RACL ligand docked at the bottom part of the receptor and at the top part of the receptor, respectively, presented slightly different distributions on 2 out of 8 MD runs.

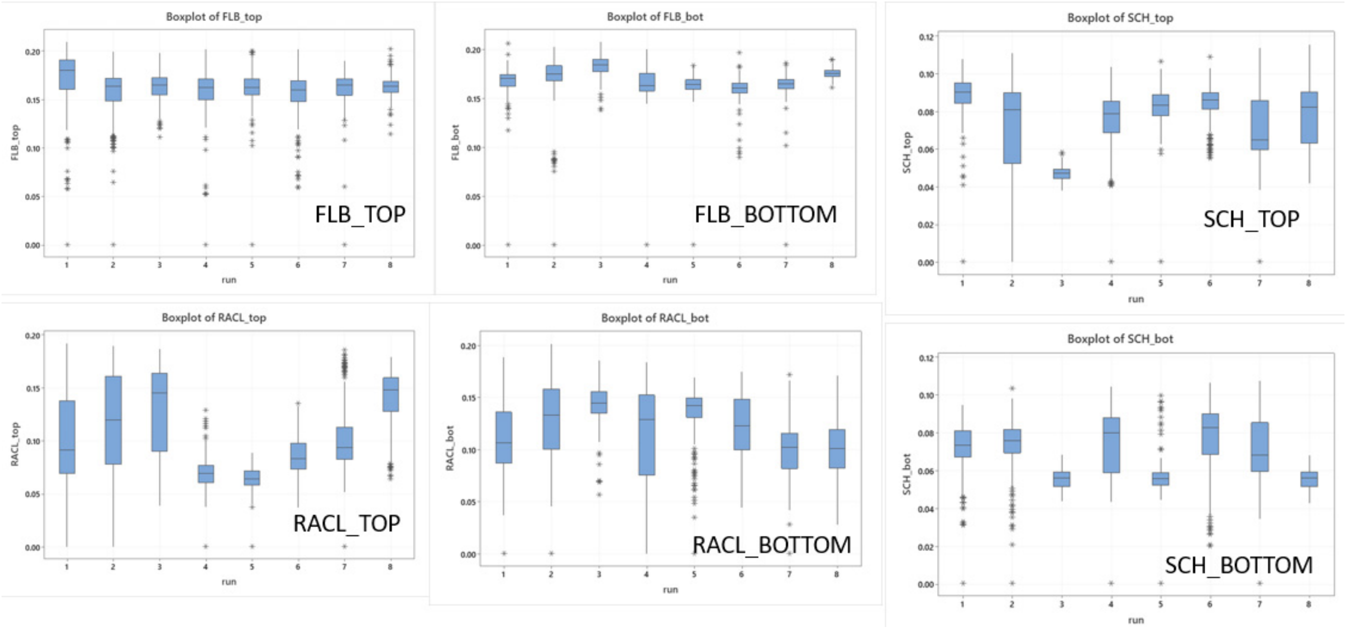

Figure S9. Boxplots of the RMSD of atom distances from the three simulation sets.

We observed different distributions again, on 2-3 out of 8 MD productions, but only for RACL and SCH ligand. We believe this variations (abnormal observations) are due to the sampling process, as a consequence of starting from slightly distinctive configurations. However, the differences between the mean values per run were approximately of 0.05 nm for RACL ligand (between runs on top and bottom positions), and 0.02 nm for SCH ligand (between runs on top and bottom positions).

Moreover, we reported higher fluctuations of RMSD profiles in the first 30 ns of MD production, particularly for RACL ligand, hence the variances in *Figure S9*.

The contour plots confirm the variations between independent productions, while the outliers (* from the boxplots) also tend to increase the estimation of variances.
